# Small-Intestinal Metastasis from Breast Cancer Presenting with Gastrointestinal Bleeding: A Case Report

**DOI:** 10.70352/scrj.cr.25-0814

**Published:** 2026-04-10

**Authors:** Ryo Hamaoka, Takahiko Kawate, Yoshiya Horimoto, Yoichi Koyama, Kayono Onishi, Kyoko Orimoto, Natsuki Uenaka, Hiroki Kusama, Yasuyuki Kagawa, Tomoya Tago, Yoshitaka Utsumi, Eiichi Sato, Hiroshi Kaise, Takashi Ishikawa

**Affiliations:** 1 Department of Breast Surgery and Oncology, Tokyo Medical University, Tokyo, Japan; 2 Department of Gastroenterology and Hepatology, Tokyo Medical University, Tokyo, Japan; 3 Department of Gastrointestinal and Pediatric Surgery, Tokyo Medical University, Tokyo, Japan; 4 Department of Anatomic Pathology, Tokyo Medical University, Tokyo, Japan; 5 Department of Pathology (Medical Research Center), Institute of Medical Science, Tokyo Medical University, Tokyo, Japan

**Keywords:** breast cancer, small intestinal metastasis, gastrointestinal bleeding, balloon-assisted enteroscopy, surgical resection

## Abstract

**INTRODUCTION:**

Gastrointestinal metastases from breast cancer are rare, especially in the small intestine, and diagnosis is often difficult due to nonspecific symptoms and limited detectability with standard endoscopy. Intestinal bleeding, although uncommon, may become clinically significant. We report a case of recurrent triple-negative breast cancer in which persistent anemia during sacituzumab govitecan (SG) therapy was attributed to small-bowel metastasis, and small-bowel evaluation using capsule endoscopy and balloon-assisted enteroscopy led to the diagnosis after standard upper and lower endoscopy failed to identify the source of bleeding.

**CASE PRESENTATION:**

A 50-year-old woman with recurrent triple-negative breast cancer underwent multiple systemic treatments before receiving sacituzumab govitecan. From treatment initiation, grade 3 anemia persisted despite transfusion support. Blood test findings indicated ongoing blood loss, including normocytic features with preserved marrow activity. Positive fecal occult blood prompted capsule endoscopy and balloon-assisted enteroscopy, which revealed a protruding jejunal lesion with active bleeding. Biopsy confirmed metastatic breast cancer. Because endoscopic hemostasis was difficult to achieve, laparoscopic partial small-bowel resection was subsequently performed. Histopathology of the resected specimen demonstrated multiple transmural lesions, many of which were suggestive of nodal metastases. Postoperatively, anemia resolved without transfusion, and SG therapy was successfully resumed after surgery.

**CONCLUSIONS:**

Small-intestinal metastasis causing bleeding is difficult to diagnose due to its nonspecific clinical presentation, and dedicated small-bowel evaluation is essential when standard endoscopy fails to identify the bleeding source.

## Abbreviations

AIHA
: autoimmune hemolytic anemia
EC
: epirubicin and cyclophosphamide
ER
: estrogen receptor
HER 2
: human epidermal growth factor receptor 2
PR
: progesterone receptor
PTX
: paclitaxel
SG
: sacituzumab govitecan

## INTRODUCTION

Distant metastases from breast cancer commonly involve the bone, lung, and liver, whereas gastrointestinal metastases are relatively uncommon.^1)^ Among these, small-bowel involvement is particularly rare. Clinical manifestations of small-bowel metastases are often nonspecific, such as perforation, obstruction, or gastrointestinal bleeding,^2,3)^ and lesions are generally difficult to detect with routine upper or lower endoscopy, making diagnosis challenging.

We report a case in which small-bowel metastasis was identified following gastrointestinal bleeding during systemic therapy for recurrent breast cancer. Because standard upper and lower endoscopy failed to detect the bleeding source, capsule endoscopy and balloon-assisted enteroscopy were required to establish the diagnosis. This case highlights the diagnostic challenges of small-bowel metastasis and underscores the importance of dedicated small-bowel evaluation in patients with unexplained anemia, which subsequently allowed appropriate local management for bleeding control.

## CASE PRESENTATION

A 50-year-old woman noticed a mass in her left breast and visited a local clinic, from which she was referred to our hospital. She was diagnosed with triple-negative (ER: 0%, PR: 0%, HER2 score: 1+, Ki67 labeling index: 50%) breast cancer (cT2N0M0, Stage IIA). Neoadjuvant chemotherapy (dose-dense EC followed by dose-dense PTX) was administered, as immune checkpoint inhibitor regimens had not yet been introduced at that time. Afterwards, curative surgery (mastectomy with sentinel lymph node biopsy) was performed. As one macrometastasis was found in the sentinel lymph node, axillary lymph node dissection was additionally performed (ypT2N1aM0, Stage IIB). Considering the residual tumor burden, adjuvant treatment with oral capecitabine was given for 6 months.

Thirteen months after surgery, metastases were detected in the pulmonary apex and hilar lymph nodes. As PD-L1 positivity was confirmed, pembrolizumab plus gemcitabine and carboplatin was initiated as treatment for recurrence and continued for 7 months until disease progression. Subsequently, due to progressive disease, the treatment regimen was sequentially switched to eribulin (3 months), trastuzumab deruxtecan (2 months), EC (4 months), and atezolizumab plus nab-paclitaxel. However, pulmonary metastases worsened and liver metastases appeared 1 month after initiating the latter regimen, leading to the introduction of SG.

**Fig. 1:**
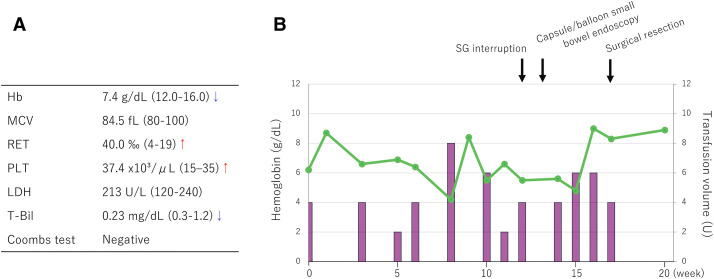
Laboratory findings at the initiation of SG therapy and clinical course thereafter. (**A**) Major laboratory findings at the initiation of SG therapy. Grade 3 anemia was present, with a normocytic MCV. Reticulocytes and platelets were mildly elevated. (**B**) Time-course of hemoglobin levels (green curve) and the clinical course, including blood transfusions (purple bars). Hb, hemoglobin; LDH, lactate dehydrogenase; MCV, mean corpuscular volume; PLT, platelet; RET, reticulocyte; SG, sacituzumab govitecan; T-Bil, total bilirubin

Mild anemia was present before treatment, and grade 3 anemia, as defined by CTCAE version 5.0, was already observed at the initiation of SG therapy, necessitating transfusion support. After SG initiation, hemoglobin levels declined further despite repeated transfusions (**Fig. 1**). **Figure 1** shows the blood test results at the start of SG and the subsequent clinical course. The anemia was normocytic, and AIHA was excluded based on a negative Coombs test. Furthermore, mild reticulocytosis and reactive thrombocytosis suggested preserved hematopoietic activity, and lactate dehydrogenase and bilirubin levels were within normal limits, making hemolysis unlikely. These findings were not compatible with simple bone marrow suppression. SG therapy was temporarily withheld at week 12 because of persistent grade 3 anemia; however, hemoglobin levels did not stabilize during this interruption. Furthermore, hemoglobin levels did not adequately increase despite repeated transfusions, raising suspicion of ongoing blood loss. Fecal occult blood testing was therefore performed and was positive.

**Fig. 2:**
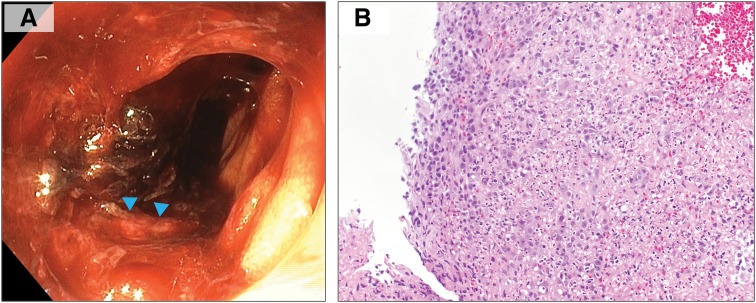
Findings of balloon-assisted enteroscopy. (**A**) Balloon-assisted enteroscopy revealed a protruding lesion with adherent blood clots in the jejunum, approximately 100–110 cm from the incisors, but hemostasis was difficult to achieve. A biopsy was conducted from the surrounding irregular mucosal area (blue arrowheads). (**B**) The biopsy specimen showed histological features similar to those of the primary breast carcinoma, consistent with metastatic involvement of the small intestine.

**Fig. 3:**
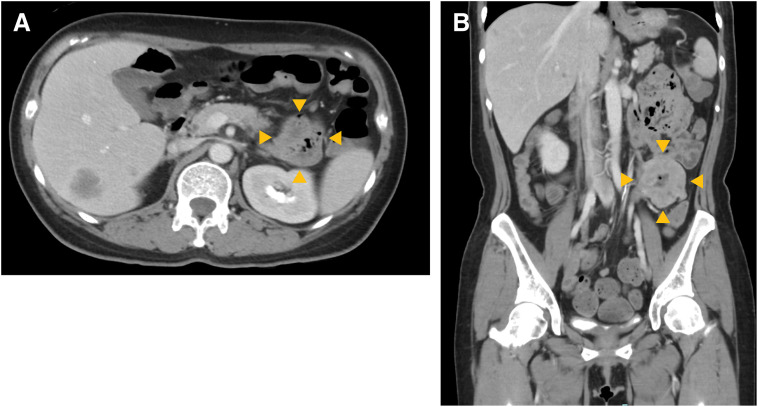
Findings of contrast-enhanced abdominal CT scan. Contrast-enhanced abdominal CT: (**A**) axial and (**B**) sagittal views. Segmental thickening of the small intestinal wall is noted (arrowheads), and hepatic metastases are also evident on the axial view.

Although upper and lower endoscopic examinations failed to identify a bleeding source, capsule endoscopy revealed a protruding lesion in the small intestine accompanied by bleeding. Subsequent balloon-assisted enteroscopy detected a 50-mm protruding lesion with adherent blood clots in the jejunum, located 100–110 cm from the incisors (**Fig. 2A**). However, hemostasis was difficult to achieve. A biopsy taken from the irregular mucosal area suspicious for a tumor revealed histological features similar to those of the primary breast carcinoma, and immunohistochemistry showed tumor cells positive for GATA3 and mammaglobin, consistent with metastatic breast cancer (**Fig. 2B**). During this period, a total of 54 units of red blood cells were transfused before the lesion was definitively identified. A contrast-enhanced CT scan performed during the same period revealed a 50-mm mass in the jejunum (**Fig. 3**). No invasion into adjacent organs such as the intestine or ureter was suspected on imaging. Laparoscopic partial resection of the small intestine was therefore planned.

**Fig. 4:**
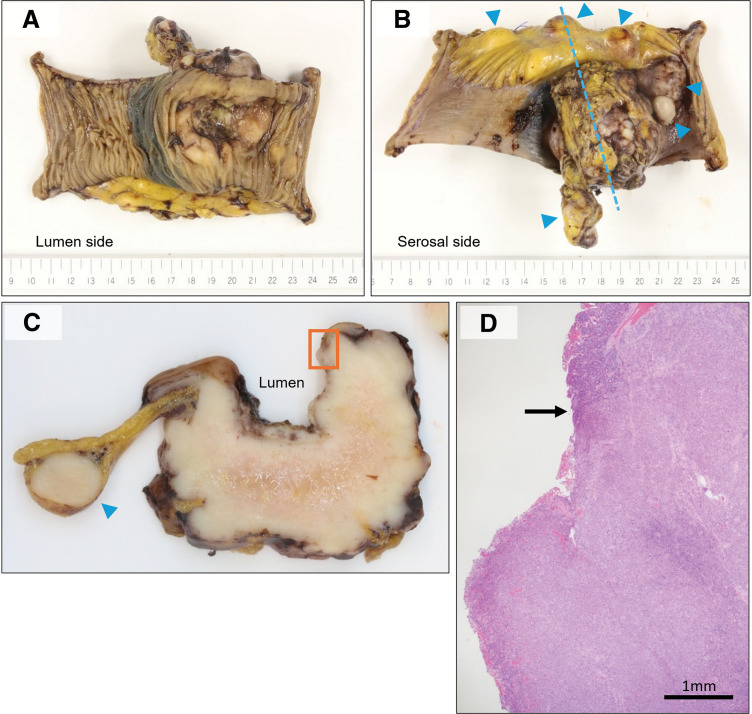
Surgical specimen of the small intestine. On both the mucosal and serosal surfaces (**A**, **B**), multiple nodular lesions were identified, measuring up to 65 mm in maximum diameter. The light blue dotted line indicates the line of section corresponding to the cut surface shown in panel C. The light blue arrowheads indicate nodular lesions apart from the main tumor. (**C**) Cross-sectioned gross view of the tumor showing that the main lesion extended through the full thickness of the small intestinal wall and formed an ulcer within the intestinal lumen. (**D**) The HE-stained image (×40) corresponds to the orange rectangular area indicated in panel C and demonstrates the boundary between the normal small intestinal mucosa and the ulcerative lesion (black arrow).

**Fig. 5:**
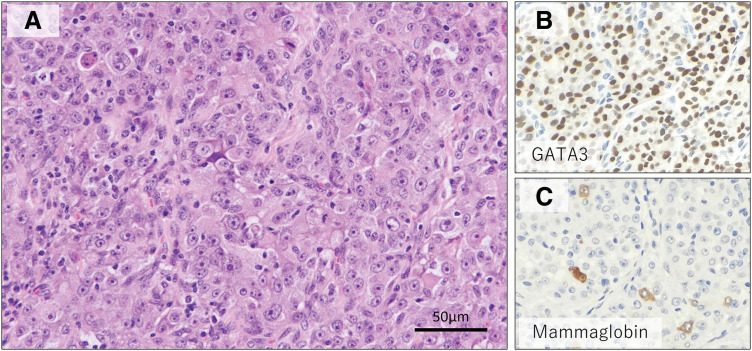
Histopathological findings of the small intestinal lesion. (**A**) High-power HE-stained view of the tumor (×200) showing densely proliferating atypical cells arranged in sheet-like fashion, with eosinophilic cytoplasm. Tumor cells were positive for (**B**) GATA3 and (**C**) partially positive for mammaglobin.

**Fig. 6:**
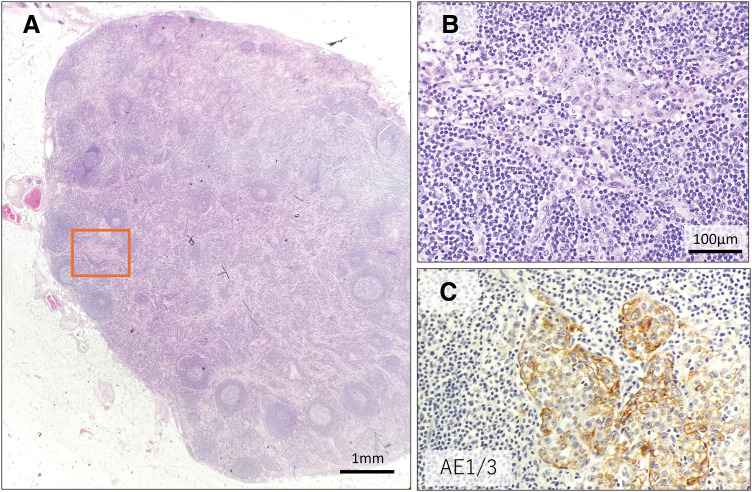
Lymph node metastasis in the surgical specimen. A mesenteric lymph node with preserved normal architecture, included in the surgical specimen, is shown (**A**, HE). A small metastatic focus was pathologically identified within this lymph node (**B**), which corresponds to the orange rectangular area indicated in panel **A**. (**C**) Cytokeratin AE1/3 staining confirms the presence of the metastatic lesion.

Intraoperatively, a single 5-mm nodule suggestive of peritoneal dissemination was observed. However, no obvious adhesion was noted around the small intestinal lesion, and a planned partial resection of the small intestine was successfully performed (operative time: 1 hour 54 minutes; blood loss: 59 mL). The surgical specimen and corresponding histological findings are shown in **Figs. 4** and **5**. Multiple nodular lesions were identified in the resected segment of the small intestine, the largest measuring 65 mm in diameter. On cut section, the main lesion involved the full thickness of the bowel wall and formed an ulcer on the luminal surface. Histologically, atypical cells with eosinophilic cytoplasm densely proliferated. The tumor cells were positive for GATA3 and mammaglobin and exhibited a triple-negative breast cancer phenotype (ER: 0%, PgR: 0%, HER2 score: 0, Ki67 labeling index: 60%). The surgical margins were negative. Several additional small nodular lesions were located within the mesentery on cut section, and histologically these were located in the subserosal adipose tissue. In one lesion, a micro-metastasis was observed within a lymph node with a preserved architecture (**Fig. 6**). Lymphovascular invasion was not clearly identified in the resected specimen.

The postoperative course was uneventful. Anemia improved without the need for postoperative transfusion, and systemic therapy with SG was resumed after surgery. The patient continues systemic treatment for recurrent breast cancer.

## DISCUSSION

Small-intestinal metastasis is uncommon, and lung cancer has frequently been reported as a primary tumor associated with this condition.^4,5)^ By contrast, among reported cases of small-intestinal metastasis from breast cancer, invasive lobular carcinoma has been the predominant histological subtype.^4)^ However, small-bowel involvement is frequently reported within the broader category of gastrointestinal metastasis, and its site-specific incidence remains unclear. Although the incidence of breast cancer is equal to or higher than that of lung cancer, the reason for the lower number of reported cases of small-intestinal metastasis in breast cancer remains unclear. Considering that the subtype with a high likelihood of small-intestinal metastasis is mainly invasive lobular carcinoma, which has a relatively low prevalence, this discrepancy may be partially explained. The present case involved invasive ductal carcinoma, which shows that small-intestinal metastasis can occur, albeit rarely, in histological subtypes other than lobular carcinoma. The temporal relationship between SG initiation and worsening anemia initially raised concern for treatment-related toxicity. However, anemia persisted even during temporary discontinuation of SG, and laboratory findings indicated preserved marrow activity, prompting further investigation for ongoing blood loss.

In patients receiving SG, grade 3 anemia may occur as a consequence of treatment-related myelosuppression. In the present case, anemia had been present before SG initiation but worsened after treatment began. However, anemia did not improve despite temporary discontinuation of SG, which was inconsistent with simple treatment-related marrow suppression. In addition, several laboratory findings were not compatible with bone marrow suppression. Reticulocytosis and reactive thrombocytosis suggested preserved hematopoietic activity, and lactate dehydrogenase and bilirubin levels were within normal limits, making hemolysis unlikely. Furthermore, hemoglobin levels did not adequately increase despite repeated transfusions, raising suspicion of ongoing blood loss. Based on these findings, fecal occult blood testing was performed and was positive, leading to further evaluation of the small intestine. The delay in definitive diagnosis was multifactorial, including the rarity of small-intestinal metastasis, initial prioritization of treatment-related causes of anemia, negative findings on CT and standard endoscopy, and temporary apparent stabilization following transfusion.

Gastrointestinal metastases may spread through lymphatic or hematogenous routes and can develop in any layer from the submucosa to the muscularis propria and serosa.^6–8)^ It is difficult to determine the exact origin of the transmural lesion that caused bleeding in this case. However, based on the macroscopic and microscopic findings of multiple small nodules in the surrounding mesentery, some of which appeared to represent lymph nodes largely replaced by tumor cells, lymphatic involvement may be suggested. Nevertheless, the metastatic route cannot be definitively determined, and hematogenous dissemination cannot be excluded. Penetration of the intestinal wall by metastatic lymph nodes causing gastrointestinal bleeding is considered relatively uncommon. Reports include a case of lymph-node metastasis from esophageal cancer penetrating the gastric wall,^9)^ as well as a case in which nodal disease from lymphoma penetrated the duodenal wall and caused arterial bleeding that was controlled by transcatheter arterial embolization.^10)^ Several cases have also been described in which metastatic mediastinal lymph nodes penetrated the trachea,^11,12)^ suggesting that metastatic lymph nodes adjacent to hollow organs may occasionally perforate these structures and cause life-threatening complications.

In advanced or recurrent breast cancer, systemic therapy is standard, and surgical resection of metastatic lesions is rarely performed. For gastrointestinal metastases, surgery is usually palliative, such as bypass procedures for obstruction.^1,13)^ However, tumor-related bleeding, as observed in the present case, may be fatal. Reports of small-intestinal metastases from lung cancer indicate that perforation and bleeding are common clinical manifestations,^5)^ and surgical resection may be effective in such cases.^14)^ Nevertheless, small-intestinal lesions are difficult to diagnose using conventional endoscopy, and capsule endoscopy or balloon-assisted enteroscopy plays an essential role.^15–17)^ In the present case, stepwise evaluation with capsule endoscopy followed by balloon-assisted enteroscopy enabled visualization, localization, and histological confirmation of the lesion, thereby establishing the diagnosis and guiding appropriate local management for bleeding control.

## CONCLUSIONS

We experienced a case of small-intestinal metastasis from breast cancer presenting with gastrointestinal bleeding. Small-intestinal metastasis should be considered in patients with metastatic breast cancer who present with persistent anemia despite negative findings on standard endoscopy. Dedicated small-bowel evaluation is essential for definitive diagnosis, and appropriate local management may facilitate continuation of systemic therapy.
